# Recurrence Risk after First Symptomatic Distal versus Proximal Deep Vein Thrombosis According to Baseline Risk Factors

**DOI:** 10.1055/s-0039-1683374

**Published:** 2019-03-11

**Authors:** Luca Valerio, Chiara Ambaglio, Marisa Barone, Mariella Ciola, Stavros V. Konstantinides, Seyed H. Mahmoudpour, Chiara Picchi, Carla Pieresca, Alice Trinchero, Stefano Barco

**Affiliations:** 1 Center for Thrombosis and Hemostasis, University Medical Center of the Johannes Gutenberg University-Mainz, Mainz, Germany; 2 Department of Internal Medicine, Fondazione IRCCS Policlinico “San Matteo,” Pavia, Italy; 3 Thromboembolic Disease Unit, Istituti Clinici Scientifici Spa SB IRCCS Maugeri, Pavia, Italy; 4 Department of Cardiology, Democritus University of Thrace, Alexandroupolis, Greece; 5 Department of Biometry and Bioinformatics, Institute for Medical Biostatistics, Epidemiology, and Informatics (IMBEI), University Medical Center of the Johannes Gutenberg University, Mainz, Germany; 6 Department of Internal Medicine, Presidio Ospedaliero “Macedonio Melloni” ASST FBF “Sacco,” Milano, Italy

**Keywords:** venous thromboembolism, pulmonary embolism, prognosis, epidemiology

## Abstract

**Background** It remains unclear whether the distal location of deep vein thrombosis (DVT) is independently associated with a lower risk of recurrence in all patients, or represents a marker of the presence and severity of provoking factors for venous thromboembolism (VTE).

**Methods** We investigated the impact of distal (vs. proximal) DVT location on the risk of developing symptomatic, objectively confirmed recurrent VTE in 831 patients with a first acute symptomatic DVT not associated with pulmonary embolism (PE), who were stratified by the presence of transient or persistent risk factors at baseline. The primary outcome was symptomatic, objectively diagnosed recurrent VTE, including proximal DVT and PE.

**Results** A total of 205 (24.7%) patients presented with a transient risk factor, 189 (22.7%) with a minor persistent risk factor, 202 (24.3%) with unprovoked DVT, and 235 (28.3%) with cancer-associated DVT. One-hundred twenty-five patients (15.0%) experienced recurrent DVT or PE. The largest relative difference between patients with distal (vs. proximal) DVT was observed in the absence of identifiable risk factors (adjusted hazard ratio [aHR]: 0.11; 95% CI [confidence interval]: 0.03–0.45). In patients with cancer, distal and proximal DVT had a comparable risk of recurrence (aHR: 0.70; 95% CI: 0.28–1.78]).

**Conclusions** The distal (vs. proximal) location of first acute symptomatic DVT represented, in the absence of any identifiable transient or persistent risk factors, a favorable prognostic factor for recurrence. In contrast, the prognostic impact of DVT location was weaker if persistent provoking risk factors for VTE were present, notably cancer.

## Introduction

Venous thromboembolism (VTE) is associated with a high risk of recurrence and death. 1 2 3 4 Age, sex, hemodynamic status at presentation, and the comorbidities represent key prognostic factors. 5 6 The location of first VTE also plays a role, as patients diagnosed with isolated distal deep vein thrombosis (IDDVT) have a lower risk of recurrence and death than those with symptomatic pulmonary embolism (PE) or proximal DVT. 7 8 9 10 11 12 This has an impact on therapeutic management: while patients with acute PE or proximal DVT invariably receive anticoagulation treatment, physicians may opt for serial imaging of the deep veins after acute IDDVT, provided that these patients do not report severe symptoms or present with major risk factors for extension. 5 13

In this perspective, it remains unclear whether the distal location of DVT is independently associated with a lower risk for recurrence in all patients, or represents a marker of the presence and severity of provoking (risk) factors. 14 15 Preliminary results from cohort studies suggest that cancer, initial burden of thrombus and degree of thrombus resolution, bilateral presentation, inpatient setting, and patient demographics might explain a significant proportion of the individual risk of recurrence in patients with first acute IDDVT. 8 10 16 17 18 19 The differential role of DVT location may therefore be less relevant in the presence of major risk factors for recurrence, which would then play as the main determinants of patients' prognosis and dictate the duration of anticoagulation; however, this question has not formally been addressed yet.

In the present analysis, we investigated the impact of distal (vs. proximal) DVT location on the risk of developing symptomatic, objectively confirmed recurrent DVT or PE in patients with a first acute symptomatic DVT not associated with PE, who were stratified by the presence of transient or persistent risk factors.

## Patients and Methods

The details of our cohort study, which retrospectively investigated the association of DVT location (IDDVT vs. proximal DVT) with DVT recurrence or survival, have been previously described. 19 In short, we included consecutive adult patients followed up at a single center between 2000 and 2012 meeting the following eligibility criteria: objective diagnosis of first symptomatic IDDVT or proximal DVT with compression ultrasound examination, no concomitant PE or prior VTE, and at least one follow-up visit. 20 In accordance with current recommendations, and upon availability of the clinical covariates, we have categorized patients according to the presence of the following risk factors 1 : transient (e.g., immobilization, recent surgery or trauma, pregnancy or caesarean section, long-haul flight) 2 ; minor persistent (e.g., autoimmune diseases, inherited thrombophilia, familiar history of VTE, congestive heart failure) 3 ; no identifiable risk factor (“unprovoked” DVT) 4 ; and cancer-associated DVT. 5 21 We did not distinguish between major and minor transient risk factors, which are viewed as a continuum in clinical practice 5 21 and, in our study population, were often concomitant to minor persistent risk factors (and therefore classified accordingly). The primary outcome was symptomatic, objectively diagnosed recurrent VTE, including proximal DVT and fatal or nonfatal PE. Recurrent events had been reviewed by two investigators based on the original reports. The secondary outcome was all-cause death.

Routine clinical care included patient education with all patients instructed to contact the center in case of signs or symptoms of recurrence. After the diagnosis of acute DVT, annual controls were scheduled and patients contacted on the same day if they missed the visit. Routine ultrasound examination of the whole leg was performed at the time of DVT diagnosis, upon anticoagulant discontinuation as a baseline reference allowing future comparisons in case of suspected recurrent events, during follow-up visits, and on suspicion of recurrence.

We accessed the center database including patient demographics and personal data. Follow-up data were extracted from source medical charts of the clinic and the institutional electronic medical record including information on admissions, consults, discharge letters, outpatient visits, and radiological data. Variable coding has been previously reported. 19 The Web site of the Local Health Authority was used for assessing patients' vital status on December 2017. Two study protocols had been developed for the primary 19 and the present analysis, and received separate approvals by the institutional Ethical Committee. Patients provided written consent for the use of clinical data at the first available follow-up visit after the first approval of the study protocol.

Descriptive analyses were performed using counts ( *n* / *N* ) and percentages for categorical data and mean/median plus adequate measures of dispersion for continuous variables. Incidence rates of recurrent VTE, expressed as number of events per 100 patient-years, were calculated for the time elapsing between first DVT and recurrence: right censoring was applied if the patient died or at the latest available follow-up visit. Cox regression models were fit to estimate hazard ratios (HRs), and corresponding 95% confidence intervals (95% CIs), for the risk of recurrent VTE after first IDDVT (vs. proximal DVT). The covariates used for calculating adjusted HRs (aHRs) were chosen based on the primary analysis; they included age, sex, recent hospitalization, and duration of anticoagulation. 19 R v.3.4.3 ( *ggplot2* , *survival* ) and SPSS v.23 (IBM, US) served for data analysis.

## Results

After screening of 4,759 medical records of patients referred to our center, 19 a total of 831 patients with first acute symptomatic DVT were included, of whom 202 had IDDVT and 629 had proximal DVT. The median age was 66 years (interquartile range [IQR], 52–76); 50.5% were women. A total of 205 (24.7%) patients presented with a transient risk factor, 189 (22.7%) with a minor persistent risk factor, 202 (24.3%) with unprovoked DVT, and 235 (28.3%) with cancer-associated DVT. Median (IQR) length of follow-up in the four groups was 4.7 (IQR 2.3–6.1), 4.6 (IQR 2.3–6.3), 4.9 (IQR 1.9–6.9), and 3.7 (IQR 0.6–6.1) years, respectively. The baseline characteristics of the study population stratified by the presence of risk factors and the location of first DVT are summarized in Table 1 . The location of cancer in patients with cancer-associated DVT is reported in Table 2 . Additional details have been previously reported. 19

**Table 1:** Baseline characteristics of the study population, number of recurrent events, and mortality rate

	Transient risk factor ( *n* = 205)		Minor persistent risk factor ( *n* = 189)		Unprovoked DVT ( *n* = 202)		Cancer-associated DVT ( *n* = 235)	
	Proximal ( *n* = 144)	Distal ( *n* = 61)	Proximal ( *n* = 139)	Distal ( *n* = 50)	Proximal ( *n* = 159)	Distal ( *n* = 43)	Proximal ( *n* = 177)	Distal ( *n* = 58)
Age (y), median (IQR)	59 (43–75)	65 (52–74)	59 (45–73)	59 (42–73)	70 (59–79)	68 (50–75)	70 (59–76)	67 (61–75)
Female sex, *n* (%)	72 (50.0)	35 (57.4)	74 (49.7)	18 (45.0)	76 (47.8)	27 (62.8)	84 (47.5)	34 (58.6)
In-hospital status at diagnosis, *n* (%)	43 (29.9)	15 (24.6)	19 (12.8)	5 (12.5)	0	0	51 (28.8)	17 (29.3)
Autoimmune disease, *n* (%)	0	0	48 (32.2)	15 (37.5)	0	0	11 (6.2)	3 (5.2)
Inherited thrombophilia, *n* (%)	0	0	50 (33.6)	13 (32.5)	0	0	14 (7.9)	2 (3.4)
Familiar history of VTE, *n* (%)	0	0	30 (20.1)	5 (12.5)	0	0	1 (0.6)	1 (1.7)
Recent long-distance travel, *n* (%)	9 (6.3)	2 (3.3)	3 (2.0)	0	0	0	0	0
Pregnancy or cesarean section, *n* (%)	7 (8.8)	0	4 (4.8) a	1 (5.9) a	0	0	0	0
Recent trauma or fracture, *n* (%)	42 (29.2)	24 (39.3)	10 (6.7) a	1 (2.5) a	0	0	5 (2.8)	3 (5.2)
Prolonged immobilization, *n* (%)	49 (34.0)	26 (42.6)	47 (31.5)	14 (35.0)	0	0	9 (5.1)	7 (12.1)
Recent surgery, *n* (%)	61 (42.4)	25 (41.0)	11 (7.4) a	4 (10.0) a	0	0	30 (16.9)	14 (24.1)
Diabetes mellitus, *n* (%)	11 (7.6)	7 (11.5)	15 (10.1)	4 (10.0)	22 (13.8)	6 (14.0)	29 (16.4)	9 (15.5)
Vascular disease, *n* (%)	32 (22.2)	13 (21.3)	29 (19.5)	10 (25.0)	54 (34.0)	13 (30.2)	23 (13.0)	14 (24.1)
Arterial hypertension, *n* (%)	39 (27.1)	20 (32.8)	47 (31.5)	16 (40.0)	75 (47.2)	18 (41.9)	68 (38.4)	28 (48.3)
Intermediate or therapeutic dosage of anticoagulant, *n* (%)	138 (98.6)	53 (89.8)	144 (98.8)	37 (94.9)	156 (98.7)	41 (95.3)	167 (96.5)	51 (91.1)
Length of anticoagulation (d), median (IQR)	212 (107–462)	83 (42–120)	302 (175–1155)	101 (43–185)	342 (103–1161)	49 (32–117)	188 (77–451)	67 (43–151)
1-y mortality, *n* (%)	9 (6.3)	2 (3.3)	2 (1.3)	1 (2.5)	9 (5.7)	1 (2.3)	58 (33.0)	22 (37.9)
10-y mortality, *n* (%)	26 (18.1)	11 (18.0)	26 (17.4)	3 (7.5)	46 (28.9)	8 (18.6)	121 (68.4)	34 (58.6)
Recurrent VTE events, *n* (%) b	22 (15.3)	4 (6.6)	26 (17.4)	4 (10.0)	36 (22.6)	2 (4.7)	25 (14.1)	6 (10.3)
PE events associated or not with DVT, *n*	2	3	3	0	9	2	2	1
Proximal DVT, *n*	20	1	23	4	27	0	23	5

Abbreviations: DVT, deep vein thrombosis; IQR, interquartile range; VTE, venous thromboembolism.

**Fig. 1:**
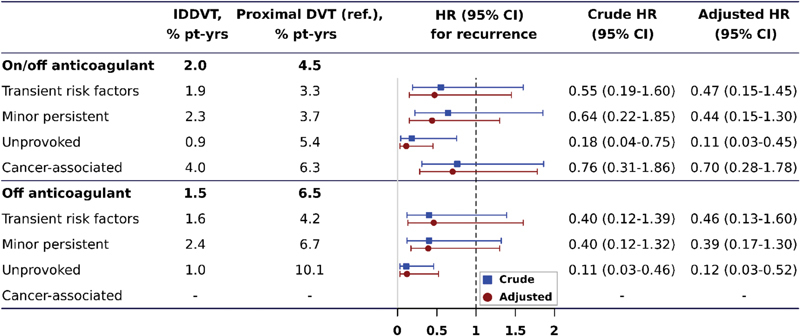
Prognostic value of distal (vs. proximal) isolated deep vein thrombosis (DVT) according to baseline provoking risk factors. Rates and hazard ratios (HR) for cancer-associated DVT patients off anticoagulants were not calculated as the mortality rate was high and vast majority of them received extended anticoagulant treatment. Adjusted HRs account for age, sex, length of anticoagulant treatment (only for events on and off anticoagulant), in-hospital status at the time of DVT diagnosis. 19 CI, confidence interval; IDDVT, isolated distal deep vein thrombosis.

a Presenting with both a transient and a minor persistent risk factor.

b Incidence rates are provided in Fig. 1 .

**Table 2:** Localization of cancer and ongoing cancer treatment in patients with proximal or distal deep vein thrombosis

	Cancer-associated deep vein thrombosis ( *n* = 235)	
	Proximal ( *n* = 177)	Distal ( *n* = 58)
Localization		
Colon, *n* (%)	35 (19.8)	5 (8.6)
Lung, *n* (%)	12 (6.8)	7 (12.1)
Breast, *n* (%)	12 (6.8)	4 (6.9)
Pancreas, *n* (%)	5 (2.8)	5 (8.6)
Leukemia, *n* (%)	33 (18.6)	6 (10.3)
Myeloproliferative, *n* (%)	6 (3.4)	1 (1.7)
Gynecological, *n* (%)	20 (11.3)	4 (6.9)
Kidney, *n* (%)	14 (7.9)	3 (5.2)
Gastric, *n* (%)	9 (5.1)	8 (13.8)
Central nervous system, *n* (%)	7 (4.0)	2 (3.4)
Liver, *n* (%)	7 (4.0)	4 (6.9)
Metastatic cancer, *n* (%)	58 (32.8)	15 (25.9)
Cancer treatment		
Chemotherapy, *n* (%)	72 (40.7)	21 (36.2)
Radiotherapy, *n* (%)	12 (6.8)	5 (8.6)
Hormonal treatment, *n* (%)	12 (6.8)	5 (8.6)

**Fig. 2:**
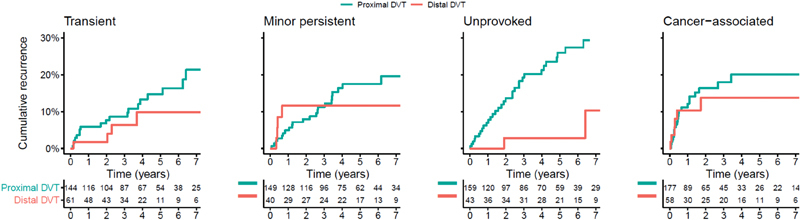
Cumulative rate of recurrent deep vein thrombosis (DVT) or pulmonary embolism in patients with first distal versus proximal DVT stratified according to baseline risk factors.

One-hundred twenty-five patients (15.0%) had recurrent symptomatic proximal DVT or PE, corresponding to overall annualized incidence rates of 2.0% in patients after IDDVT and 4.5% after proximal DVT. The annualized rates of recurrence in patients stratified by DVT location and baseline risk factors are presented in Fig. 1 . The largest relative difference between patients with distal and proximal DVT was observed in the absence of identifiable risk factors (adjusted HR [aHR]: 0.11; 95% CI: 0.03–0.45) after adjustment for age, sex, and length of anticoagulant treatment. Similar results were obtained if only events off anticoagulation were considered ( Fig. 1 ). The impact of DVT location was less prominent in the presence of cancer (aHR: 0.70 for distal vs. proximal DVT [95% CI: 0.28–1.78]). No firm conclusions could be drawn for patients with transient (0.47 [95% CI: 0.15–1.45]) and minor persistent (aHR: 0.44; [95% CI: 0.15–1.30]) provoking risk factors, due to the lack of statistical power. The cumulative recurrence in patients with distal and proximal DVT stratified according to baseline risk factors is depicted in Fig. 2 .

**Fig. 3:**
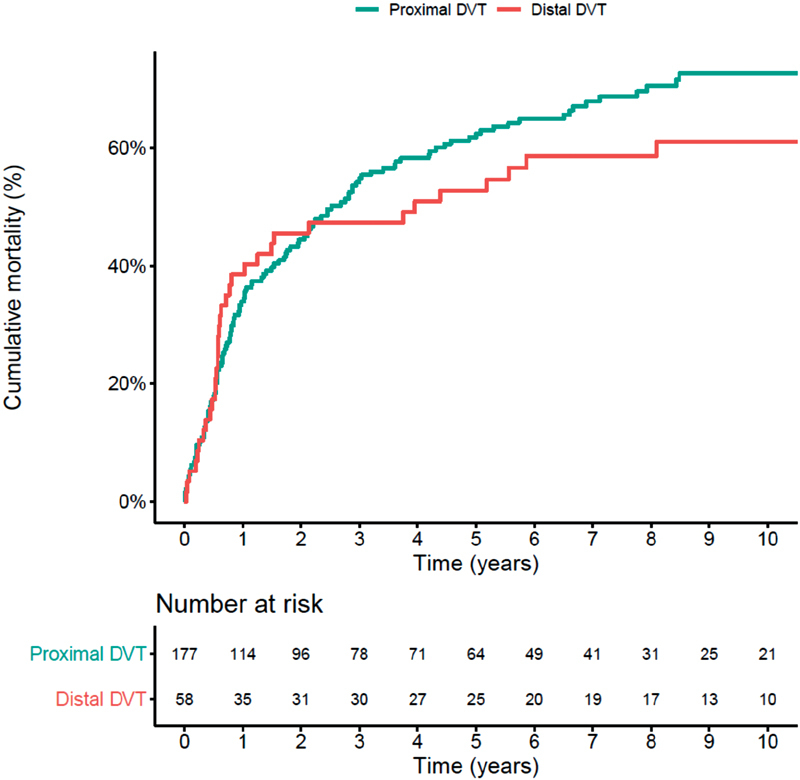
Cumulative mortality after first distal versus proximal cancer-associated deep vein thrombosis (DVT).

Since cancer patients were characterized by the highest 1-year mortality (33.0% after proximal DVT and 37.9% after distal DVT) and 10-year mortality (68.4% after proximal DVT and 58.6% after distal DVT), in this group we used Cox regression models adjusted for different potential confounders to assess the impact of DVT location also on all-cause mortality ( Table 3 ). At univariate analysis, HR (95% CI) for distal (vs. proximal) DVT was 0.67 (95% CI: 0.26–1.72). In the fully adjusted model accounting for age, sex, in-hospital status at diagnosis, presence of metastasis, cardiovascular or autoimmune disease, and recent surgery/trauma, HR for distal (vs. proximal) DVT was 1.02 (95% CI: 0.69–1.50). The cumulative mortality in patients with distal and proximal DVT is depicted in Fig. 3 .

**Table 3:** Impact of distal (vs. proximal) deep vein thrombosis location on mortality in cancer patients

	Hazard ratio (95% confidence interval)
Model 1: univariate	0.67 (0.26–1.72)
Model 2: adjusted for age and sex	0.87 (0.60–1.27)
Model 3: adjusted for age, sex, in-hospital status, metastasis, cardiovascular disease	0.96 (0.67–1.41)
Model 4: adjusted for age, sex, in-hospital status, metastasis, cardiovascular disease, recent surgery or trauma, autoimmune diseases, immobilization	1.02 (0.69–1.50)

## Discussion

The decision to continue anticoagulation for an extended period after DVT depends on the estimated risk of progression or recurrence after diagnosis and, thereafter, after discontinuing anticoagulation. 22 The results of our analysis confirm that the risk of recurrence is substantial not only in patients with a first unprovoked *proximal* DVT event (annualized rate of 10.1% after anticoagulant discontinuation), but also in those with a proximal DVT caused by transient (4.2%) or minor persistent risk factors (6.7%). 23 24 These rates are comparable to that described in a post hoc analysis of VTE patients enrolled in the Einstein CHOICE trial. 23 Consistently, a recent population-based study conducted in Denmark showed that patients with first unprovoked VTE had similar 6-month risk of recurrence compared with those with non–cancer-provoked VTE. 25 These data challenge the notion of tailoring anticoagulation to the individual patient on the basis of categorization of transient and persistent risk factors other than cancer, also because a large proportion of patients may present with both transient and persistent risk factors, or with multiple persistent risk factors. 26

On the other hand, we found that patients with a first episode of IDDVT in the absence of identifiable risk factors were at truly low risk of developing long-term recurrence (1.0% per year after anticoagulant discontinuation). This rate is lower than the one observed in the OPTimisation de l'Interrogatoire pour la Maladie thromboEmbolique Veineuse (OPTIMEV) study (3.8%), 10 but comparable to the results of the Austrian Study on Recurrent Venous Thromboembolism (AUREC) study (1.7%). 8 The differences may be explained, at least in part, by the longer follow-up time in AUREC (10 years) and in the present analysis (∼4.5 years) compared with OPTIMEV (3 years), leading to a dilution of the initial peak of recurrence usually observed after anticoagulant discontinuation. 22

Our results indirectly support the hypothesis that not all patients with IDDVT may even require initial anticoagulant therapy due to their negligible risk of progression or recurrence, as suggested by the results of the CACTUS trial, in which low-risk outpatients with IDDVT were randomized to receive either low-molecular-weight heparin or placebo. 27 In contrast, based on our data only patients with unprovoked IDDVT appeared at a truly low risk of recurrence after standard course of anticoagulation with an annualized rate of 1.0%, whereas the presence of additional risk factors for recurrence doubled this risk. The long-term risk of recurrence and death was comparable between patients with distal and proximal cancer-associated DVT, with aHR of 0.70 (95% CI: 0.28–1.78) and 1.02 (95% CI: 0.69–1.50), respectively. Our results confirmed prior reports showing that the short-term risk of recurrence is substantial in patients with cancer-associated IDDVT. 17 28 These findings support the view that decisions on anticoagulation should be primarily based on the assessment of individual risk factors rather than categorization by location, and that distal versus proximal DVT location appears relevant only in patients at low risk in whom no provoking factors are identified. 26

Limitations of our analysis include confounding by indication (IDDVT patients were often treated for shorter periods and with lower anticoagulant doses 19 ) and wide confidence intervals of the estimates. Moreover, the classification of patients according to baseline risk factors was done retrospectively. Finally, the relatively low count of events recorded in this study did not allow us to adjust for other important variables which may confound the association between DVT location and outcomes.

## Conclusions

The distal (vs. proximal) location of first acute symptomatic DVT represented, in the absence of any identifiable transient or persistent risk factors, a favorable prognostic factor for recurrence. This observation supports the decision to abstain from extended anticoagulant therapy after unprovoked IDDVT. In contrast, the prognostic impact of DVT location was weaker or absent if persistent provoking factors for VTE are present, notably cancer. These results should be taken into account when tailoring the duration of anticoagulant treatment in patients diagnosed with acute symptomatic DVT.
